# The safety and efficacy of PD-1 inhibitors in patients with advanced cancers and HIV/AIDS in China

**DOI:** 10.3389/fonc.2023.1248790

**Published:** 2023-09-19

**Authors:** Yu Xiong, Pingzheng Mo, Yajun Yan, Shan Wang, Ke Zhuang, Zhiyong Ma, Xiaoping Chen, Liping Deng, Yong Xiong, Di Deng, Yongxi Zhang

**Affiliations:** ^1^ Department of Radiation and Medical Oncology, Zhongnan Hospital of Wuhan University, Wuhan, China; ^2^ Hubei Key Laboratory of Tumor Biological Behaviors, Zhongnan Hospital of Wuhan University, Wuhan, China; ^3^ Hubei Clinical Cancer Study Center, Zhongnan Hospital of Wuhan University, Wuhan, China; ^4^ Department of Infectious Diseases, Zhongnan Hospital of Wuhan University, Wuhan, China; ^5^ Centre of AIDS Prevention and Cure, Zhongnan Hospital of Wuhan University, Wuhan, China; ^6^ ABSL-III Laboratory at the Center for Animal Experiment, Wuhan University, Wuhan, Hubei, China

**Keywords:** HIV, cancer, camrelizumab, immunotherapy, PD-1

## Abstract

Purpose-Immunotherapy has revolutionized cancer therapy, becoming the standard of care for various malignancy treatments. Human immunodeficiency virus (HIV) patients, however, are an underserved group with limited access to clinical trials and cancer therapy. This study was to evaluate the safety and efficacy of programmed cell death 1 (PD - 1) inhibitors in patients with advanced cancer and HIV/acquired immunodeficiency syndrome (AIDS). Methods and Materials-We performed a prospective, open-label, nonrandomized, phase 1 single center study. Patients with advanced cancer and HIV/AIDS received the treatment of PD - 1 inhibitors (camrelizumab, 200 mg, administered intravenously every 3 weeks), along with combination antiretroviral therapy (cART) for HIV. Results-Sixteen participants (12 men and 4 women; median age, 46.5 (29 - 78) years) were enrolled; 1 had non - Hodgkin lymphoma (NHL), and 15 had non - AIDS - defining cancers. Safety was observed over 130 cycles of treatment with camrelizumab. Most treatment-emergent adverse events at least possibly attributed to camrelizumab were grade 1 or 2, including reactive cutaneous capillary endothelial proliferation (RCCEP) (9 participants), hearing loss (1 participant), hypophysitis (1 participant). 3 participants experienced hemorrhage due to poor performance status. HIV was controlled in all participants. Best tumor responses included 3 complete response, 5 partial response, 2 stable disease, and 6 progressive disease. The 2 years progression-free survival (PFS) was 67.0% (95% CI: -0.05, 0.00) and overall survival (OS) was 55.3% (95% CI: -0.05, 0.01) for the 16 patients who had received camrelizumab. Conclusions-This study demonstrates that camrelizumab treatment in patients with advanced cancers and HIV/AIDS was feasible and the clinical outcomes were acceptable.

## Introduction

The first case of acquired immunodeficiency syndrome (AIDS) in China was reported in 1985 by Peking Union Medical College Hospital in Beijing. Since then, the human immunodeficiency virus (HIV)/AIDS pandemic in China has grown rapidly, particularly in the last ten years. A total of 110,000 persons in China were newly infected in 2020, making up 1.04 million HIV - positive individuals (1). Compared to the general population, people living with HIV (PLWH) have a higher chance of acquiring cancer. Historically, cancers that arise in the context of HIV have been divided into two categories: cancers that, when present, provide an AIDS diagnosis (AIDS - defining malignancies, or ADMs), and cancers that do not necessarily signal AIDS (non - AIDS - defining malignancies, or NADMs) (2). Combination antiretroviral therapy (cART) was introduced around 1996, which led to a 75 - 80% decrease in the incidence of various ADMs (3), largely as a result of a decline in the prevalence of profound immunodeficiency. An rising percentage of all malignancies in PLWH in North America are NADMs, such as lung cancer, Hodgkin lymphoma, anal cancer, and oropharyngeal cancer (4, 5). Similar trends have been observed in Europe, Australia, and the Asia-Pacific region (6, 7). PLWH still have less access to conventional cancer therapy despite the rise in cancer incidence (8).

Checkpoint inhibitors have transformed the way many malignancies are treated in recent years. A checkpoint molecule called programmed cell death 1 (PD - 1) inhibits T cell antigen receptor signaling, including that of CD8^+^effector T cells (9). A humanized, selective IgG4 - κ monoclonal antibody against PD - 1 known as camrelizumab (Jiangsu Hengrui Pharmaceuticals Co, Ltd) demonstrated anticancer efficacy in a variety of tumors (10–12). The participation in clinical trials, particularly those testing novel immunotherapy drugs, has consistently been denied to these HIV - positive cancer patients (8). These exclusions are made because there is a risk of disease reactivation or flare - up, there are elevated immune - related toxicities, and there may be insufficient efficacy in these patient subgroups (13)

The associated immune - related adverse events (irAEs) well characterized with all immune checkpoint blockers are one of the major obstacles to developing anti - PD - 1 as a strategy for an HIV cure (13). Compared with the others irAEs, such as immune ‐ related pneumonitis and colitis, the incidence of reactive cutaneous capillary endothelial proliferation (RCCEP) caused by camrelizumab is obviously high. Studies showed that incidences of RCCEP ranged from 60% to 90% (10–12). About RCCEP, most occurred after the first cycle of camrelizumab (2 - 4 weeks), predominantly on the face and trunk, and disappeared spontaneously after camrelizumab discontinuation (1 - 2 months). Interestingly, previous studies showed that the occurrence of RCCEP induced by camrelizumab is associated with the better prognosis (10–12). It seems that HIV - infected individuals have similar tolerance to checkpoint inhibitors, when compared to the non - HIV infected population (14). However, the safety profile of anti - PD - 1 treatment in PLWH is uncertain

Despite the fact that immunotherapy appears to be generally well tolerated in HIV patients with cancer, strong evidence is still needed to prove the treatment’s safety and effectiveness generally, and particularly in actual clinical oncology practice (15). Furthermore, through affecting HIV latency and HIV - specific immunity, several cancer immunotherapies may potentially alter HIV persistence (16). In this study, we analyzed the safety and efficacy of PD - 1 inhibitors in the treatment of advanced cancer in HIV/AIDS patients in order to provide clinical data supporting immunotherapy in HIV/AIDS patients with cancer

## Materials and methods

### Study population

HIV/AIDS patients with advanced cancers who received camrelizumab at the Zhongnan Hospital of Wuhan University from September 2020 to August 2022, were included in the study. Inclusion criteria included patients diagnosed with malignant tumor by histopathological examination, patients failing in previous therapies due to the side effects of chemotherapy, and disease progression or relapse. Exclusion criteria included patients with uncontrolled opportunistic infection, autoimmune disease, or abnormal heart, liver, and kidney function.

All patients in this study received immunotherapy (camrelizumab, 200 mg, administered intravenously every three weeks), along with cART (Bictegravir/Emtricitabine/Tenofovir alafenamide tablets) for HIV and cotrimoxazole to prevent pneumocystis carinii pneumonia (PCP). This study was approved by the Medical Ethics Committee of Zhongnan Hospital of Wuhan University (No. 2021064). It was performed in accordance with the principles of the Declaration of Helsinki. All patients provided written informed consent.

### Observation and data collection

Baseline data, including gender, age, complications, pathologic type of cancers, previous antitumor therapy, cART regimen, and CD4^+^ T lymphocyte count before immunotherapy were collected. Laboratory tests, including blood routine, liver function, myocardial enzyme, thyroid hormone, adrenocortical hormone, hepatitis B surface antigen, hepatitis C antibody, and HIV viral load were performed before and during treatment. Hepatitis B virus (HBV) - DNA and hepatitis C virus (HCV) - RNA were further tested if hepatitis B surface antigen and hepatitis C antibody were tested positive. HBV - DNA of less than 30 IU/mL, HCV - RNA of less than 50 IU/mL, and HIV - RNA of less than 20 copies/mL were considered negative. Tumor staging was performed according to the corresponding tumor staging standards. Lymphoma was staged using Ann Arbor staging standards while liver cancer, cervical cancer, anal cancer, lung cancer, gastric cancer and laryngeal cancer were staged using TNM staging standards.

During follow - up visits, the side effects of PD - 1, HIV status, and tumor responses were monitored. The Eastern Cooperative Oncology Group performance status (ECOG PS) score was used to assess the physical status of the patients. Adverse events (AEs) were evaluated and graded by the NCI - CTCAE version 5.0. The HIV viral load was tested every 3 weeks for the first 9 weeks after treatment and every 3 months thereafter. Imaging examinations were performed every 6 weeks to objectively assess the tumor responses. Solid tumor responses were assessed by Response Evaluation Criteria In Solid Tumors v1.1 (17), lymphoma responses by the refined Lugano classification lymphoma response criteria (18).

### Statistical analysis

The safety population included all patients who received at least 3 dose of camrelizumab (n = 16). Baseline characteristics were tabulated and summarized. All observed AEs were tabulated and treatment-emergent AEs (TEAEs) at least possibly attributed to camrelizumab, all serious AEs (grades 3 - 4) were recorded. Tumor responses were evaluated using standard tumor - specific criteria. Statistical analyses were performed using SAS (version 9.4).

## Results

### Patient characteristics

Sixteen patients between September 2020 and August 2022 were enrolled in this study (**Table 1**). The median age was 46.5 (29 - 78) years; 12 (75%) patients were men and 4 (25%) were women. All the patients were Han Chinese. 7 (44%) patients had an ECOG PS of 0, 5 (31%) had an ECOG PS of 1, 2 (12%) had an ECOG PS of 2, and 2 (12%) had an ECOG PS of 3. One (6%) had AIDS - defining cancers, refractory diffuse large B cell lymphoma (DLBCL). Fifteen (94%) had non - AIDS - defining cancers, refractory Hodgkin lymphoma (3), hepatocellular cancer (HCC) (3), recurrence invasive cervical cancer (ICC) (3), non - small cell lung cancer (NSCLC) (2), small cell lung cancer (SCLC) (1), gastric adenocarcinoma (1), laryngeal cancer (1), and anal cancer (AC) (1). One HCC patient was infected with HBV and HCV simultaneously. Patients were heavily pretreated; the median number of prior systemic therapies was 4 (range, 0 - 8), 4 (27%) had received previous radiation therapy and 2 had received four and seven interventional treatments, respectively.

**Table 1:** The characteristics of 16 patients with advanced cancer and HIV infection.

case	gender	age	type of tumor	PS	Clinical stages	Previous lines of therapy	CD4 counts	Complicate diseases	Cycle of PD-1 inhibitor	Other antitumoral regimen
1	male	34	HL	0	IIa	ABVD × 6 cycles	301	No	11	NA
2	female	57	HL	0	IIIb	ABVD × 4 cycles	177	Hpertension	8	NA
3	male	42	HL	0	IIb	ABVD × 4 cycles	170	No	8	NA
4	male	29	DLBCL	0	IIEa	R-EPOCH × 4 cycles, RR × 4 cycles	182	No	16	lenalidomide× 6 months
5	male	48	HCC	1	T3N1M0	NA	40	Hepatitis B, hepatitis C, cirrhosis	15	lenvatinib × 5 months
6	female	45	ICC/ SCC	1	T4N2M1	Cisplatin × 6 cycles plus radiotherapy	86	No	20	apatinib × 6 months
7	male	78	NSCLC/ADC	1	T3N1M0	EP × 4 cycles	425	Hpertension	3	NA
8	male	40	AC/SCC	2	T4N2M1	EP × 2 cycles plus radiotherapy	165	Rectourethral fistula	3	NA
9	male	47	HCC	2	T3N2M0	Interventional therapy × 4 times	360	Hepatitis C cirrhosis, esophageal and gastric varices	3	lenvatinib × 6 months
10	female	43	ICC/ SCC	3	T4N2M1	Paclitaxel plus lobaplatin × 4 cycles, radiotherapy	287	vesico-vaginal fistula	3	NA
11	female	55	ICC/ SCC	0	T2N1M0	Cisplatin × 6 cycles plus radiotherapy	181	Pericardial and pleural effusion	6	albumin paclitaxel plus bevacizumab× 4 cycles
12	male	68	gastric adenocarcinoma	0	T2N2M0	Operation, SOX × 6 cycles	246	No	8	apatinib × 6 months
13	male	46	HCC	1	T3N1M0	Interventional therapy × 7 times plus Sorafenib	402	Hepatitis B cirrhosis	14	lenvatinib × 6 months
14	male	62	SCLC	3	T4N2M1	NA	294	No	3	EP × 2 cycles
15	male	66	laryngeal cancer	1	T2N1M0	NA	174	No	3	DC × 2 cycles
16	male	46	NSCLC	0	T2N1M0	NA	644	Chronic hepatitis C	6	EP × 6 cycles

ABVD, bleomycin, oncovin, doxorubicin, dacarbazine; AC, anal cancer; ADC, adenocarcinoma; CCEP, cutaneous capillary endothelial proliferation; DLBCL, diffuse large B cell lymphoma; EP, etoposide, carboplatin; HCC, hepatocellular cancer; HL, Hodgkin’s lymphoma; ICC, invasive cervical cancer; NA, not available; NSCLC, non-small cell lung cancer; PD, progressive disease; PR, partial response; PS, performance status; R-EPOCH, rituximab, etoposide, cyclophosphamide, adriamycin, oncovin, prednisone; RR, rituximab, lenalidomide; SCC, squamouscell cancer; SD, stable disease; SOX, oxaliplatin, tegafur.

### Treatment

Safety was observed over the course of 130 cycles in 16 patients. The median number of cycles was 6 (range, 3 - 20). At the time of analyses, 7 patients continued to receive therapy. 10 patients received targeted therapy or chemotherapy in combination with camrelizumab, but the targeted therapy did not exceed 6 months and the chemotherapy did not exceed 4 cycles.

### Safety outcomes

**Figure 1:**
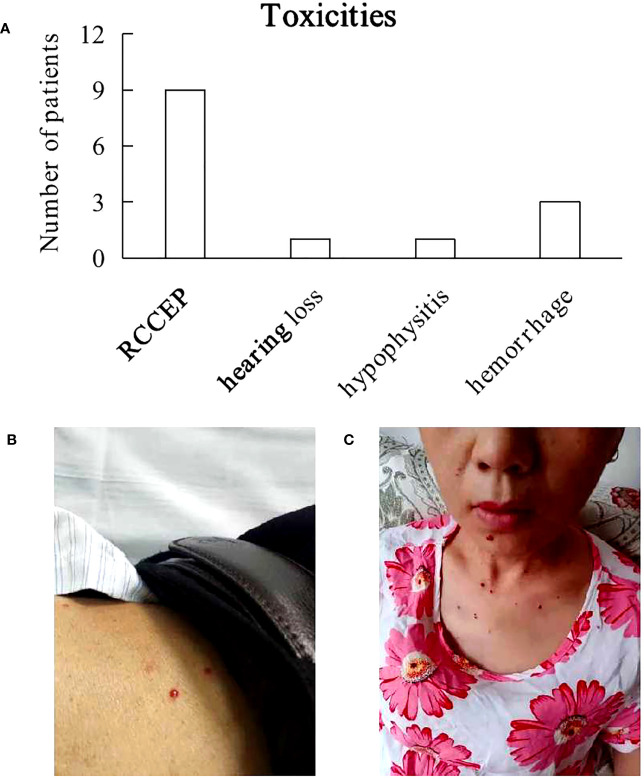
Toxicities. **(A)** Most treatment-emergent adverse events at least possibly attributed to camrelizumab were grade 1 or 2, including reactive cutaneous capillary endothelial proliferation (RCCEP), hearing loss, hypophysitis. 3 patients experienced hemorrhage due to poor performance status. **(B, C)** Photos of 2 patients with RCCEP.

TEAEs at least possibly attributed to camrelizumab that occurred is included in **Figure 1A**. After 2 cycles of camrelizumab treatment, nine (56%) patients developed grade 1 - 2 of RCCEP successively (**Figures 1B, C**). The NSCLC patient presented grade 1 RCCEP and bilateral hearing loss after 3 cycles of camrelizumab. RCCEP was resolved and the hearing in the left ear was recovered after camrelizumab treatment was terminated, however, the hearing loss in the right ear did not improve significantly. One Hodgkin lymphoma patient presented grade 1 RCCEP and hypophysitis, and recovered after discontinuing camrelizumab and steroids therapy. Three patients were suspended from 3 cycles of camrelizumab due to massive hemorrhage. One ICC patient complicated with a vesico-vaginal fistula. Bladder hemorrhage occurred after 3 cycles of camrelizumab, and inferior gluteal artery embolization was performed after the failure with hemostatic drugs therapy. This patient gave up all treatments for cancer and died 3 months later. One HCC patient was complicated with hepatitis C cirrhosis and esophageal and gastric varices. Upper gastrointestinal bleeding occurred suddenly after 3 cycles of camrelizumab, which was confirmed by gastroscopy and immediately treated with endoscopic hemostasis. This patient was then treated with lenvatinib thereafter while continuing to take sofosbuvir and velpatasvir for 24 weeks. This patient was in a stable condition. The AC patient had a large perianal ulcer with a rectourethral fistula. The ulcer continued to bleed after 3 cycles of camrelizumab and the bleeding was stopped by inferior gluteal artery embolization. This patient was then treated with sorafenib, and cancer progressed slowly.

### CD4 and HIV monitoring

**Figure 2:**
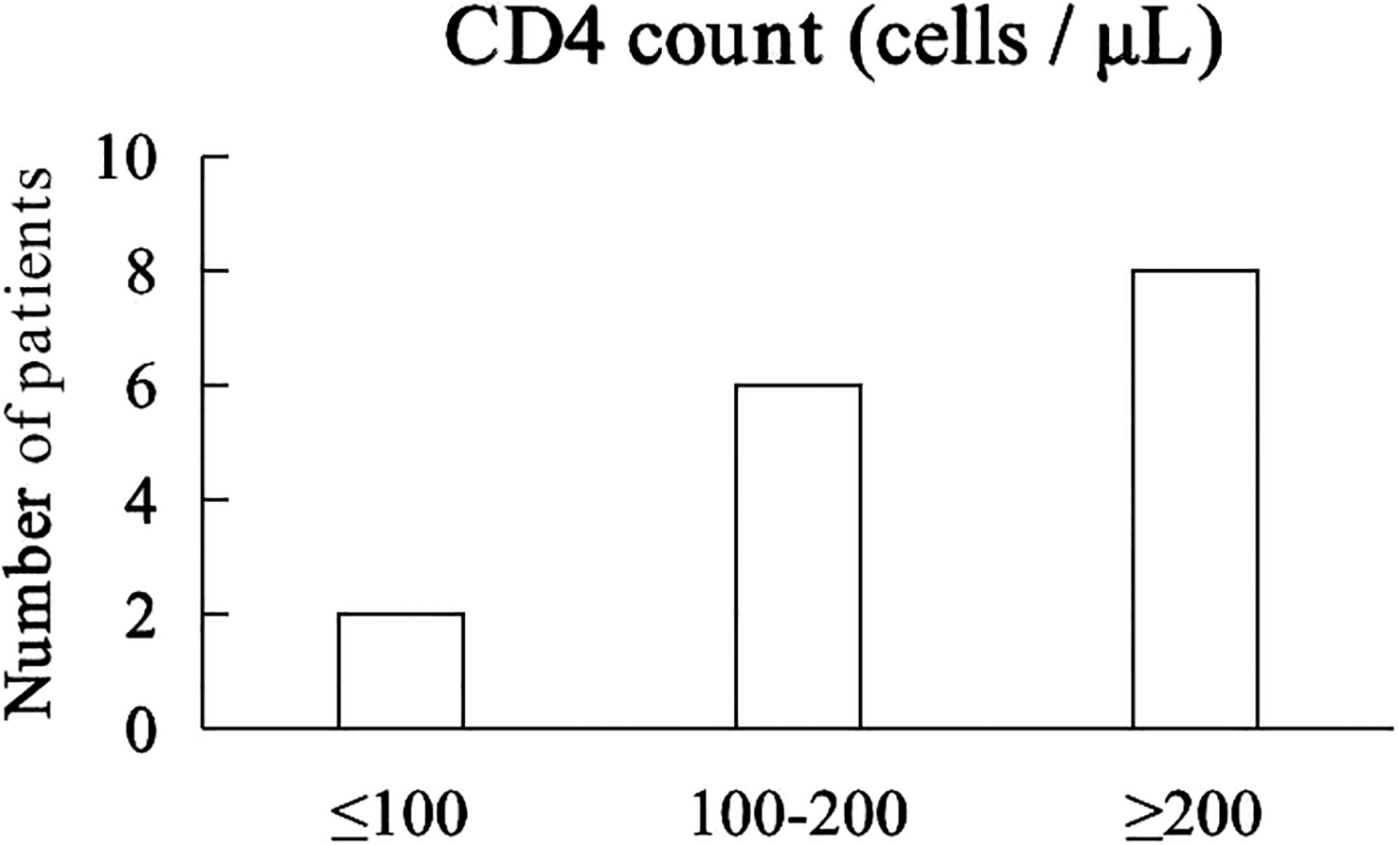
CD4^+^ T cell counts of 16 patients.

The median CD4 count was 214 cells/μL (40 - 644 cells/μL). 2 patients with CD4^+^ T lymphocyte count less than 100 cells/µL and 6 patients with CD4^+^ T lymphocyte count between 100 and 200 cells/µL, 8 patients with CD4^+^ T lymphocyte count greater than 200 cells/µL (**Figure 2**). Prior to receiving immunotherapy, 15 patients who were treated with cART were tested negative for HIV viral load, and their HIV - RNA was tested negative throughout camrelizumab, while one patient who did not start cART had high levels of HIV viremia (147,000 copies/mL), received cART and camrelizumab almost simultaneously had significantly lower level of HIV - RNA after 4 weeks and the negative level of HIV - RNA and HBV - DNA were continued even after 8 weeks of immunotherapy.

### Responses

**Figure 3:**
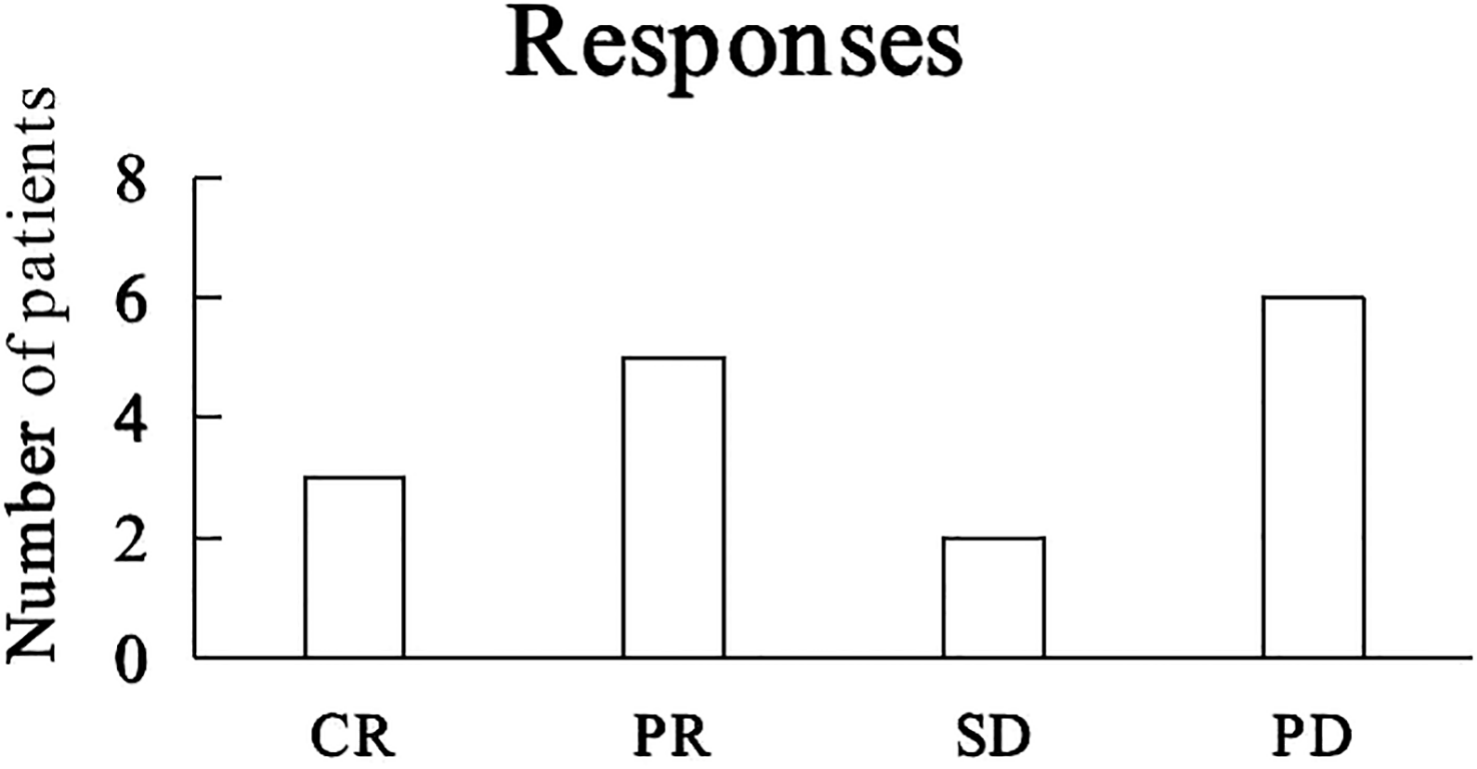
Tumor responses to camrelizumab. CR: complete response, PR: partial response, SD: stable disease, PD: progressive disease.

Best tumor responses included complete response (3 Hodgkin lymphomas, 3 patients), partial response (1 HCC, 1 ICC, 1 gastric adenocarcinoma, 1 laryngeal cancer, 1 NSCLC, 5 patients), stable disease for 13 months for 1 HCC patient and 21 months for 1 ICC patient, and progressive disease (1 DLBCL, 1 NSCLC, 1 SCLC, 1 AC, 1 HCC, 1 ICC, 6 patients) (**Figure 3**).

### Survival

**Figure 4:**
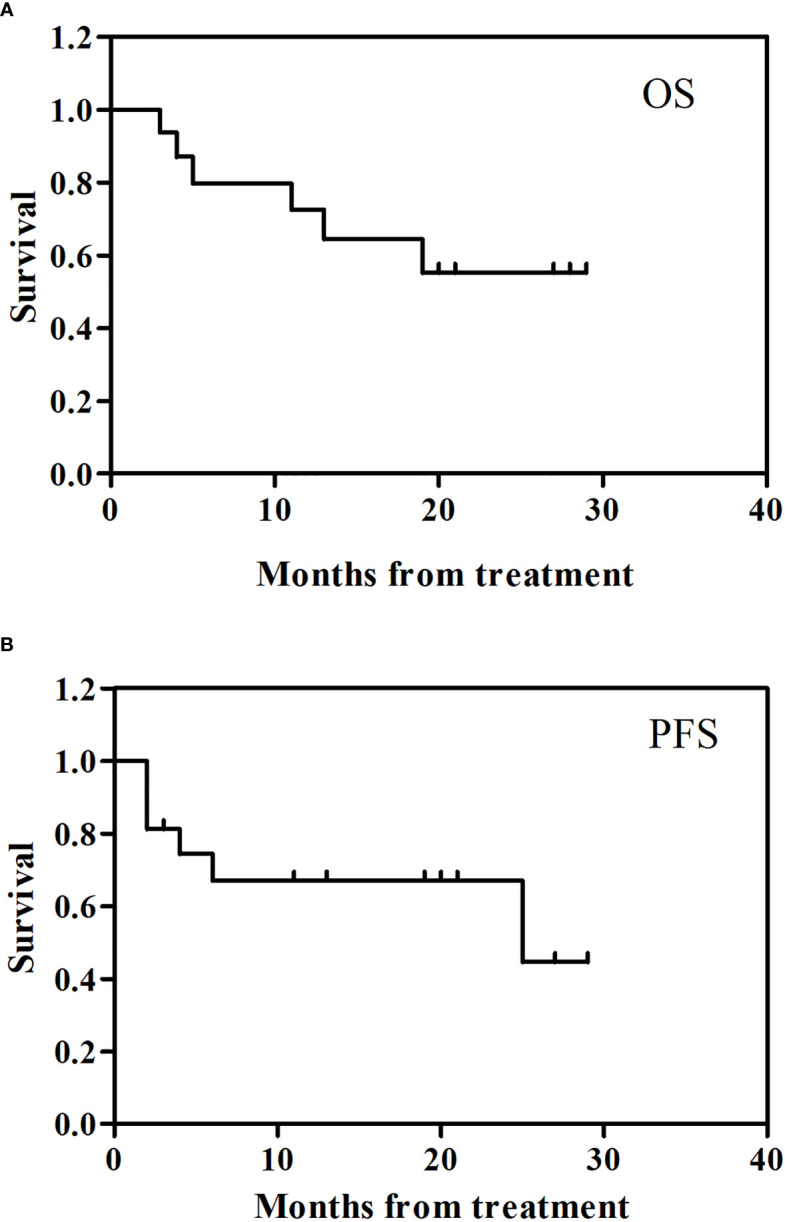
Clinical outcomes. **(A)**: overall survival (OS) and **(B)**: progression-free survival (PFS).

**Figure 4** showed the follow up clinical outcome following camrelizumab. The 2 year overall survival (OS) rate for the 16 patients enrolled was 55.3% (95% CI: -0.05, 0.00) (**Figure 4A**) and the 2 year progression-free survival (PFS) was 67.0% (95% CI: -0.05, 0.00) (**Figure 4B**).

## Discussion

Applying immunotherapy to a patient who also has cancer and HIV/AIDS is a significant accomplishment. The safety and efficacy of anti - PD - 1 and anti - PD - L1 antibodies in HIV - infected individuals with cancer have been examined in two prospective trials thus far (19, 20), and other retrospective and case series have corroborated (21, 22). To the best of our knowledge, this is the first prospective trial to be conducted in China examining the viability and safety of using PD - 1 inhibitors in the treatment of HIV/AIDS and advanced cancer patients. The most frequent adverse events (irAEs) was RCCEP, which was reported by 9 (56%) of the patients. Three patients in this research experienced significant hemorrhages, and embolization was a successful hemostatic procedure. The findings indicated that patients with advanced cancer and HIV infection, particularly those with ulcers or nasal passages, needed to be aware of the possibility of bleeding during camrelizumab treatment.

HIV^+^ patients with melanoma, lung cancer, Merkel cell carcinoma, Kaposi sarcoma, and other cancers have received a variety of immune checkpoint inhibitor regimens to date, including pembrolizumab, nivolumab, ipilimumab, ipilimumab and pembrolizumab, atezolizumab, avelumab, and durvalumab (23). Patients responded to the therapy in a variety of ways, but generally speaking, neither the number of CD4^+^ cells nor the level of plasma viral load decreased or dramatically increased. Evidence of safety and efficacy in Kaposi sarcoma, NHL, lung cancer, and liver cancer were found in a prospective phase 1 study of pembrolizumab in PLWH with a CD4 count > 100 cells/µL and advanced malignancy (19). Six patients with a CD4^+^ T lymphocyte count between 100 and 200 cells/µL and two patients with a CD4^+^ T lymphocyte count below 100 cells/µL in our research both responded well to immunotherapy and experienced no serious side effects. Immunotherapy may be safe for patients with low CD4^+^ T lymphocyte counts, according to our research, which demonstrated no association between the therapeutic benefit and CD4^+^ T lymphocyte count. In addition, 9 patients in our research received cART before beginning PD - 1 inhibitor, and tests for HIV - RNA remained negative throughout PD - 1 inhibitor therapy. Another patient who had cART and PD - 1 inhibitor nearly concurrently had considerably decreased HIV - RNA levels after four weeks, and the negative HIV - RNA level persisted even after eight weeks of immunotherapy. Following PD - 1 inhibitor therapy, HIV - RNA was consistently negative, demonstrating that PD - 1 inhibitors did not activate HIV, which is consistent with multiple previous clinical trials and retrospective investigations reports (21).

With the advent of immunotherapy in recent years, the treatment paradigm for a number of malignancies has undergone a significant shift. The majority of immune checkpoint inhibitor clinical studies completed to date, however, have either excluded patients with poor PS or persistent illnesses like HBV and HCV or have accumulated a very small percentage of aged individuals. On the safety and effectiveness of immunotherapy in poor PS patients, there is minimal information available. Only four prospective studies with PS 2 patients have been reported to date (24–27). Given that PS 2 patients are already a pretty varied group on their own, it is challenging to draw particular conclusions from these trials because they predominantly comprised a combination of elderly patients and/or PS 0 - 1 individuals with comorbidities (e.g., renal impairment, chronic viral hepatitis). Patients with poor PS brought on by cancer and tumor load may benefit from therapy, especially if rapid response can be established. For patients who have poor PS owing to comorbidities, however, the risk - benefit ratio is different and safety is prioritized. Due to advanced malignancy and serious comorbidities (cirrhosis and esophagogastric varices in one patient, extensive local ulcers in two patients), two PS 2 patients and one PS 3 patient in our study experienced major bleeding.

Due to worries about viral reactivation, anxieties about increased toxicity, and probable ineffectiveness in certain patient categories, individuals with HBV and HCV have often been excluded from most cancer trials. Although HBV infections are now considered chronic illnesses, HCV infections are even curable because to modern antiviral medications. Unfortunately, there is little published information on the safety of immunotherapy for patients with advanced malignancies and HBV infections. According to a few case studies and one retrospective case series, immunotherapy in some patients with chronic or prior HBV infection may result in HBV reactivation. Pu et al. recently conducted a comprehensive review that includes 14 papers (8 case reports, 4 case series, and 2 trials) (28) for HBV - and HCV - positive cancer patients receiving immunotherapy. Although hepatitis reactivations may occur, rigorous monitoring and early antiviral treatment are advised, the authors came to the conclusion that immunotherapy use is regarded safe and effective in individuals with chronic HBV/HCV. In our trial, 4 (25%) patients had HBV and/or HCV infections; however, early antiviral medication administered during camrelizumab immunotherapy prevented any reactivation of hepatitis.

The so - called special populations include elderly patients. One of the primary causes of the increasing cancer incidence with age is likely immune senescence (29). It is currently unknown, nevertheless, how immunological senescence could impact the effectiveness and security of anticancer therapy. A pooled survival analysis encompassing 2824 individuals recruited into four studies contrasting immunotherapy with docetaxel was conducted by the FDA in the United States (30). Grades 3 to 4 AEs were reported in 23% of patients aged ≥ 75 years who received the anti - PD - 1/PD - L1 arm as opposed to 49% of individuals aged ≥ 65 years and 47% of patients aged < 65 years. In addition, there were no significant differences in any of the AEs of specific interest (hypothyroidism/elevated thyroid stimulating hormone, colitis, hepatitis, pneumonia, or other). Thus, there was no indication of a more severe toxicity in the group of patients ≥ 75 years old, however it should be remembered that, once again, the proportion of extremely elderly patients concluded up being small. Given that they were clearly underrepresented, these patients were also most likely carefully chosen. After receiving camrelizumab medication, one patient in our research, who was 78 years old, had grade 1 RCCEP with bilateral hearing loss. After stopping the usage of camrelizumab, RCCEP was resolved and the hearing was somewhat returned.

### Limitations

The study did not have enough patients with any given tumor to accurately estimate response rates or to compare response rates with those of people with the same cancers but no HIV. In addition, CD4^+^ T lymphocyte count was not monitored regularly.

## Conclusions

Treatment with camrelizumab is feasible in patients with advanced tumors and HIV/AIDS. Patients with advanced cancer and HIV/AIDS may consider cancer therapies with immune checkpoint inhibitors even in special populations (elderly patients and pre-existing chronic HBV/HCV infections), but priority is given to safety for patients who have poor PS due to comorbidities.

## Data availability statement

The raw data supporting the conclusions of this article will be made available by the authors, without undue reservation.

## Ethics statement

This study was approved by the Medical Ethics Committee of Zhongnan Hospital of Wuhan University (No. 2021064). It was performed in accordance with the principles of the Declaration of Helsinki. All patients provided written informed consent. The studies were conducted in accordance with the local legislation and institutional requirements. Written informed consent for participation was not required from the participants or the participants’ legal guardians/next of kin in accordance with the national legislation and institutional requirements.

## Author contributions

All authors contributed to the study conception and design. Material preparation were by YuX, PM, YY. data collection were by YuX, SW, KZ, ZM, XC, and analysis were performed by LD, YoX. The first draft of the manuscript were written by DD and YZ and all authors commented on previous versions of the manuscript. All authors contributed to the article and approved the submitted version.
